# Efficacy and safety analysis of AKT inhibitor in triple-negative breast cancer: A meta-analysis and systematic review

**DOI:** 10.1097/MD.0000000000039347

**Published:** 2024-08-30

**Authors:** Minghao Yang, Chunxi Wang, Guoping Chen, Haowen Zhang, Junlong Lin

**Affiliations:** a The First Affiliated Hospital of Hainan Medical University, Haikou, Hainan, China; b Chinese PLA General Hospital, Beijing, China.

**Keywords:** AKT inhibitors, triple-negative breast cancer, capivasertib, ipatasertib, AZD5363, GDC0068

## Abstract

**Objective:**

To determine the clinical benefit of monotherapy with AKT inhibitors in patients diagnosed with triple-negative breast cancer (TNBC).

**Methods:**

A systematic search was conducted in PubMed, Embase, and Cochrane Library for articles reporting treatment with AKT inhibitors in TNBC. The primary endpoint was progression-free survival and overall survival (OS). Secondary endpoints included the clinical benefit rate (CBR, included the proportion of patients with complete response, partial response, and stable disease), overall response rate (ORR, included the proportion of patients with complete response and partial response), all drug-related adverse events (AEs), and ≥3 grade drug-related grade AE.

**Results:**

We included 723 patients from 5 studies and observed a pooled progression-free survival of 0.80 (95% CI: 0.62–1.02; The Grading of Recommendations, Assessment, Development, and Evaluations [GRADE] assessment: moderate certainty) and OS of 0.7 (95% CI: 0.50–0.99; GRADE assessment: high certainty) in TNBC patients treated with AKT inhibitors. Regarding clinical benefit rate and overall response rate were 1.21 (95% CI 0.85–1.73; GRADE assessment: moderate certainty) and 1.26 (95% CI 0.91–1.73; GRADE assessment: low certainty). Only OS had a statistical difference. For the odd ratio of all grade AE and ≥3 grade AE in the therapeutic process was counted and pooled, 4.34 (95% CI 1.33–14.14; GRADE assessment: moderate certainty) and 1.76 (95% CI 1.28–2.41; GRADE assessment: moderate certainty), respectively.

**Conclusions:**

AKT inhibitors showed slightly better efficacy in the treatment of TNBC. However, further studies are needed to evaluate its long-term safety and optimal regimen, and caution should be exercised in patients with coexisting gastrointestinal disorders. The clinical characteristics of the patients and the choice of drugs should be considered on an individual basis.

## 1. Introduction

Breast cancer (BC) is the most prevalent cancer among women and the second leading cause of cancer-related mortality worldwide.^[1]^ BC is categorized into 3 major subtypes based on molecular markers: estrogen receptor, progesterone receptor, and human epidermal growth factor receptor 2 (HER2): hormone receptor-positive, HER2-positive, and triple-negative breast cancer (TNBC). TNBC accounts for approximately 15 to 20% of all breast carcinomas.^[2]^ Compared with hormone receptor-positive BCs, TNBC has a worse prognosis, with over 50% of patients experiencing a relapse in the first 3 to 5 years post-diagnosis, and the median overall survival (OS) based on current therapies is 10.2 months.^[3]^

The PI3K/AKT/mTOR pathway, which is frequently mutated in TNBC, operates via a ligand (such as insulin or an insulin-like growth factor) that binds to a cell membrane receptor. Upon activation by an extracellular ligand, stimulates PI3K (phosphatidylinositol (3,4,5)-trisphosphate kinase). Activated PI3K triggers phosphorylation of PIP2 at the 3 position of the inositol ring to produce PIP3. This process recruits 2 protein kinases to the plasma membrane through their pleckstrin homology interaction domains (PH domains), AKT (also known as protein kinase B (PKB)) and PDK1 (phosphoinositide-dependent protein kinase 1). Once drawn to the cell membrane, AKT is phosphorylated by mTORC2 (mTOR complex 2) at Ser473, altering the conformation of AKT and allowing its phosphorylation at Thr308 by PDK1. Activated AKT initiates the phosphorylation of target proteins from the cell membrane, detaches them from the cell membrane, and phosphorylates other target proteins in the cytosol and cell nucleus. The phosphorylation of these target proteins stimulates cell survival, growth, and proliferation.^[4]^ AKT, a crucial downstream effector of the PI3K pathway,^[5]^ has emerged as a promising therapeutic target in TNBC. Several AKT inhibitors have been formulated and evaluated in clinical trials for TNBC, exhibiting varying efficacy and safety profiles.

In this meta-analysis and systematic review, we aimed to perform a comprehensive evaluation of the efficacy and safety of AKT inhibitors in TNBC. AKT inhibitors can be categorized into 2 main subgroups: ATP competitors that compete with ATP to associate with Akt kinase at the ATP binding site and include capivasertib (AZD5363), GSK2110183, GSK690693, and ipatasertib (GDC0068), and allosteric inhibitors that target the PH domain and prevent the migration of AKT to the plasma membrane where activation by upstream kinases occurs, thus locking AKT in an inactive form.^[6]^ This analysis primarily included capivasertib and ipatasertib, 2 of the most advanced AKT inhibitors. Capivasertib and ipatasertib are highly selective oral ATP-competitive small-molecule inhibitor of all 3 AKT isoforms. They are being developed for the treatment of cancers in which PI3K/AKT pathway activation may be relevant for tumor growth or therapeutic resistance, and have demonstrated PI3K/AKT pathway inhibition in preclinical studies.^[7,8]^ By synthesizing data from multiple clinical trials, we aimed to provide a comprehensive overview of current evidence regarding the use of AKT inhibitors in TNBC treatment. This analysis will contribute to the understanding of the potential role of AKT inhibitors in the management and guide future clinical research and practice.

## 2. Material and methods

### 2.1. Inclusion and exclusion criteria

To be included in this meta-analysis, the RCTs had to fulfill the following criteria: (1) all patients were diagnosed with TNBC by histopathology. (2) Type of intervention: Akt inhibitors as first-line therapy vs other routine therapies; (3) primary endpoint: median progression-free survival rate (PFS), overall survival (OS), overall response rate (ORR), clinical benefit rate (CBR), adverse event (AE) incidence rate, and ≥3 grade AE incidence rate.

The exclusion criteria were as follows: (1) the trial size or study population was unclear and (2) studies with duplicate publications or overlapping data. (3) Studies without available data were included. (4) Excluding non-English literature.

### 2.2. Search strategy and study identification

**Figure 1.**
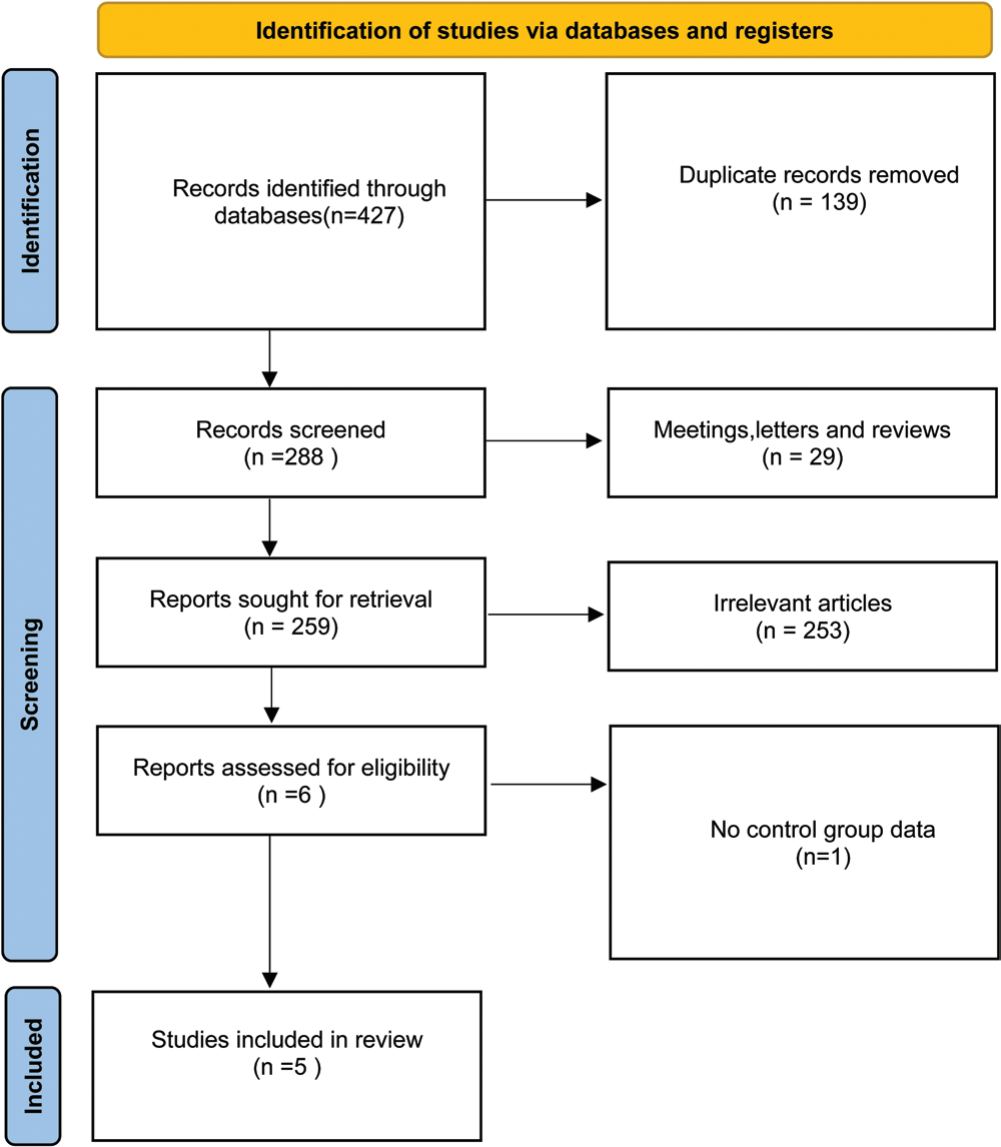
The diagram of study selection and identification.

Eligible studies were identified by a systematic literature search of PubMed, Embase, and Cochrane databases, with no date restrictions up to November 20, 2023 (Web of Science was not included because PubMed contains almost all of its literature). The search strategy was carried out using the following keywords: “triple negative breast cancer”, “ipatasertib”, “GDC-0068”, “capivasertib”, and “AZD5363”. Specific keywords and free text terms were combined using Boolean operators. The detailed process is illustrated in Figure 1. A systematic literature search was carried out independently by 2 authors, and any discrepancies were resolved by discussion with a third author. This systematic review and meta-analysis was conducted according to the Preferred Reporting Items for Systematic Reviews and Meta-Analyses (PRISMA) guidelines.^[9]^ This study is registered with PROSPERO registration number CRD42023485340; the full protocol is freely available on the PROPSPERO website.

### 2.3. Statistical analysis

Meta-analysis was performed using RevMan 5.4 and Stata 18.0. PFS and OS were expressed as hazard ratio (HR) and 95% confidence interval (CI), and the incidence of ORR, CBR, and ≥3 grade AE were calculated using odds ratios (OR) and 95% CI. The χ^2^ test was used to analyze the heterogeneity among the studies. There was no statistical heterogeneity among the studies if *P* > .1 and I^2^ < 50%. A fixed-effects model was used to analyze heterogeneity. Otherwise, a random-effects model was used. Statistical significance was set at *P* < .05.

### 2.4. Certainty of evidence

The Grading of Recommendations, Assessment, Development, and Evaluations (GRADE)^[10]^ approach has been used to rate the overall certainty or quality of the evidence for each outcome on the basis of risk of bias, imprecision, indirectness, inconsistency, and publication bias. We used the GRADE approach to assess the certainty of evidence.

## 3. Results

### 3.1. Characteristics of included studies

**Figure 2.**
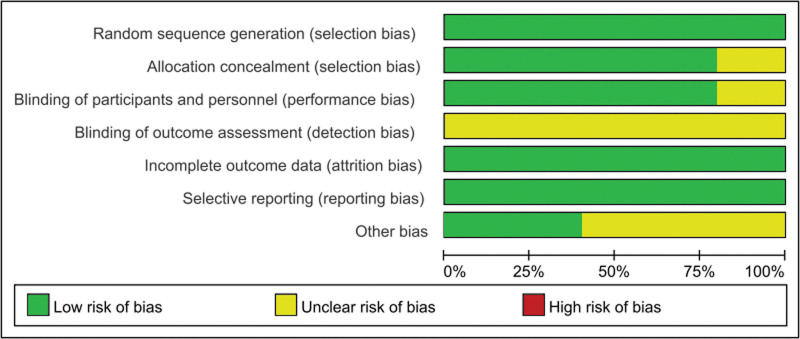
Quality assessment of individual study. Green represents low risk of bias, yellow represents unclear risk of bias, and red represents high risk of bias.

**Figure 3.**
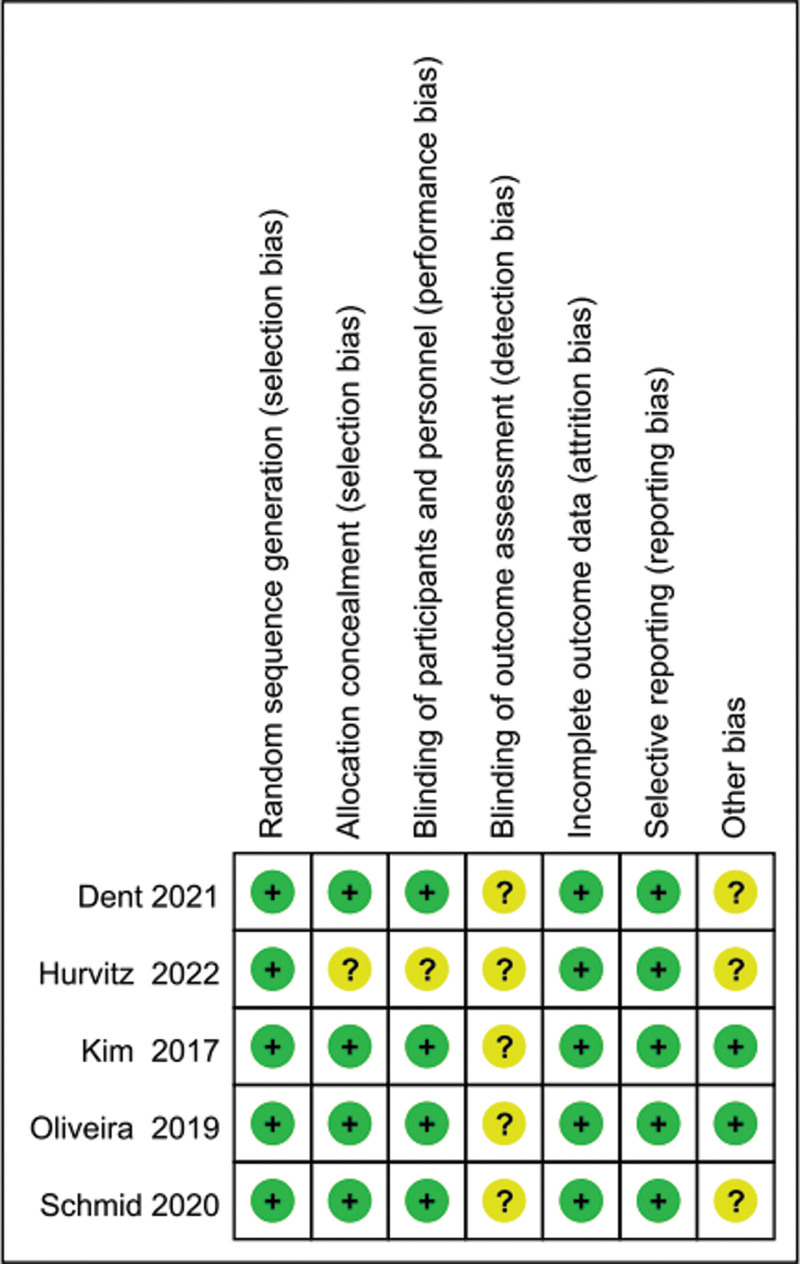
Quality assessment of individual study. Green represents low risk of bias, yellow represents unclear risk of bias, and red represents high risk of bias.

A total of 427 articles were screened, and 5 studies were finally included,^[11–15]^ which involved 405 and 318 participants in the intervention and control groups, respectively (Fig. 1). The treatment adopted in the intervention groups comprised placlitaxel or atezolizumab plus AKT inhibitors (capivasertib and ipatasertib), while the comparators were placebo plus placlitaxel or capecitabine. The characteristics of the included trials are shown in Table 1. The respective bias of individual studies was acceptable (Figs. 2 and 3), while publication bias was not evaluated because of the insufficient number of adopted studies.

**Table 1:** The diagram of study selection and identification.

Author	Year	Median follow-up time (month)	No. of patients		Treatment		Outcome
			Intervention	Control	Intervention	Control	
Schmid	2020	18.2	70	70	Capivasertib + Paclitaxel	Placebo + Paclitaxel	①②③④⑤⑥
Kim	2017	Intervention 16 and control 19	62	62	Ipatasertib + Paclitaxel	Placebo + Paclitaxel	①②③④⑤⑥
Hurvitz	2022	10.8	29	24	Atezolizumab + Ipatasertib	Capecitabine	①②③⑤
Dent	2021	8.3	168	87	Ipatasertib + Paclitaxel	Placebo + Paclitaxel	①③④⑤
Oliveira	2019	NA	76	75	Ipatasertib + Paclitaxel	Placebo + Paclitaxel	③⑤⑥

① mPFS: median progression-free survival; ② mOS: median overall survival; ③ ORR: objective response rate; ④ CBR: clinical benefit rate; ⑤ ≥3 grade adverse events; ⑥ all adverse events.

### 3.2. Efficacy

**Figure 4.**
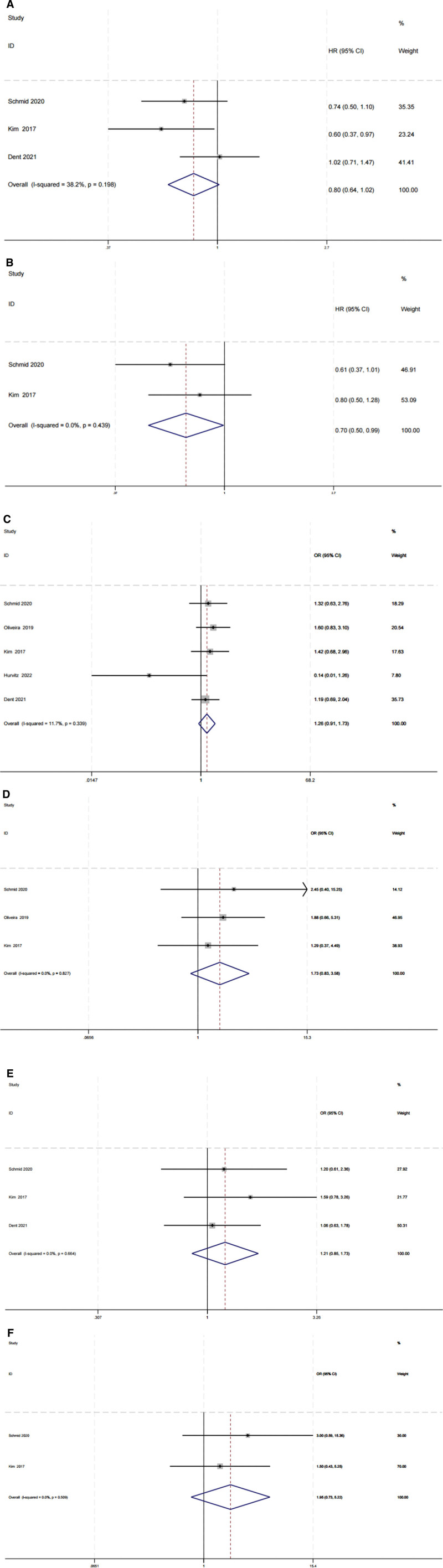
(A) Progression-free survival. (B) Overall survival. (C) Objective remission rate. (D) Objective remission rate of PIK3CA/AKT1/PTEN-altered subgroup. (E) Clinical benefit rate. (F) Clinical benefit rate of PIK3CA/AKT1/PTEN-altered subgroup.

For PFS, 3 publications involving 519 patients were included in the comparative analysis. Using a fixed-effects model with acceptable heterogeneity (I^2^ = 38.2%, *P* = .198), HRs pertaining to PFS from randomized controlled trials were extracted and aggregated. In these trials, AKT inhibitors (vs placebo plus paclitaxel therapy arm) demonstrated no significant statistical meaning. As evidenced in Figure 4A, the result revealed that the pooled HR for PFS was 0.80 (95% CI: 0.62–1.02; GRADE assessment: moderate certainty).

In terms of OS, 2 studies containing 264 participants were included in the analysis. Based on a fixed-effects model (I^2^ = 0, *P* = .439), the combination of paclitaxel and AKT inhibitors likely represents the optimal treatment, exhibiting superior efficacy compared to paclitaxel-only regimens. Figure 4B indicated that the pooled HR for OS was 0.7 (95% CI: 0.50–0.99; GRADE assessment: high certainty).

Nevertheless, our analysis included a study showing no superior median PFS (mFPS) and median OS (mOS) for AKT inhibitors compared to capecitabine, at 1.7 and 10.8 months respectively, which could not be pooled because of the absence of HR for PFS and OS.

Data for the ORR were available for analysis from 5 clinical trials involving 713 patients. The overall ORR was 1.26 (95% CI 0.91–1.73; GRADE assessment: low certainty), as determined by the fixed-effect model (I^2^ = 11.7%, *P* = .339). The overall ORR for the PIK3CA/AKT1/PTEN-altered subgroup, based on 3 clinical trials, was 1.73 (95% CI 0.83–3.58; GRADE assessment: high certainty), as per the fixed-effect model (I^2^ = 0, *P* = .827) (Figs. 4C and 4D).

Three studies with 517 participants were included in the analysis. Based on a fixed-effects model (I^2^ = 0, *P* = .664), AKT inhibitors appeared to be a superior option. The overall CBR was 1.21 (95% CI 0.85–1.73; GRADE assessment: moderate certainty). For the PIK3CA/AKT1/PTEN-altered subgroup, the CBR was 1.95 (95% CI 0.73–5.22; GRADE assessment: high certainty) (Figs. 4E and 4F).

### 3.3. Safety

**Figure 5.**
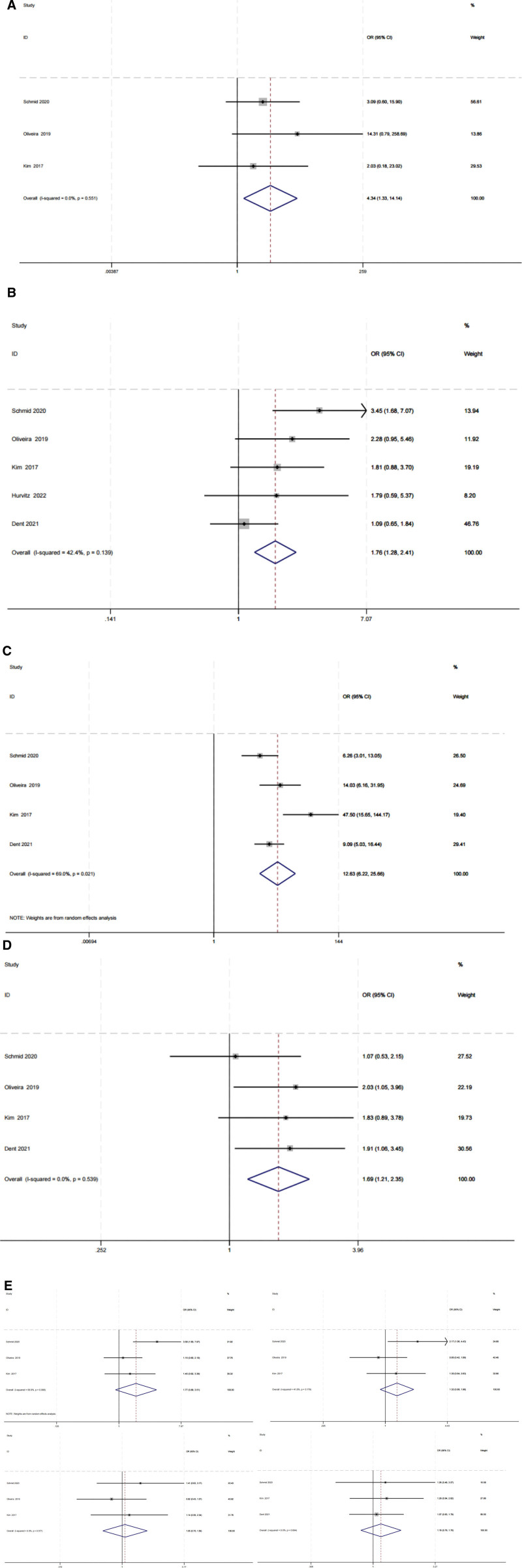
(A) All grade adverse events. (B) ≥3 Grade adverse events. (C) Diarrhea. (D) Nausea. (E) Rash, fatigue, neuropathy, and alopecia.

All considered studies reported serious adverse drug events. The OR of all grade AEs and ≥3 grade AE during the therapeutic process was calculated and pooled, yielding 4.34 (95% CI 1.33–14.14; GRADE assessment: moderate certainty) and 1.76 (95% CI 1.28–2.41; GRADE assessment: moderate certainty) respectively (Figs 5A and 5B). The results indicated that the test group experienced significantly more severe AEs than the control group. After collating data on the 6 most commonly occurring AEs (diarrhea, fatigue, nausea, rash, neuropathy, and alopecia), we observed that while the AKT inhibitors in combination with paclitaxel or atezolizumab demonstrated slightly better improvement than the placebo therapy arm, patients in the new combination treatment arm experienced a higher incidence of diarrhea and nausea. The differences were statistically significant based on the random-effects model analysis (Figs. 5C and 5D). No statistically significant differences were found in the OR for fatigue, rash, neuropathy, and alopecia (Fig. 5E).

## 4. Discussion

Our meta-analysis, which included 723 patients from 5 studies, demonstrated that targeted AKT inhibitors had only a slight advantage over the control group in patients with TNBC.

Dysregulation of the PI3K/AKT/mTOR pathway is recognized as a marker of cancer development and gene alterations associated with this pathway are frequently observed in TNBC.^[16]^ Overactivation of the PI3K/AKT/mTOR pathway supports the hypothesis that targeting this signaling pathway may provide a beneficial therapeutic strategy. Consequently, numerous AKT inhibitors have been developed for cancer treatment over the past few years. Although TNBC lacks effective treatment options, chemotherapy has been the primary treatment for advanced TNBC. However, with the emergence of several innovative therapeutic agents, we are entering a new era of TNBC management. Among them, a meta-analysis conducted by Han^[17]^ compared the efficacy of novel treatment regimens for TNBC, and found that chemotherapy plus AKT inhibitors may be the best treatment choice, which provides a reference basis for our meta-analysis. However, the results of our study are contrary to those of previous studies, which may be due to the small sample size.

In addition, it is worth noting that 2 studies^[12, 15]^ did not align with other results, with even poorer efficacy noted, and the outcome of Hurvitz study was in direct contrast to that of our meta-analysis. Owing to the inability to collect the HR of PFS and OS in this study, we could not include them in the forest map. However, he reported that the median mPFS and mOS were 1.7 months and 10.8 months respectively, both lower than those of the control group in his study. We believe that there are the following reasons: first, the drugs used in this study are different from those used in other studies, which may produce different therapeutic effects; second, the sample size of this study was small, which increases the probability of bias; and finally, some studies have shown that AKT inhibitors can relieve drug resistance. It is possible that our study demonstrated that AKT inhibitors have a better effect on paclitaxel than atezolizumab, but larger studies are needed to confirm this. Even with studies yielding different results, AKT inhibitors generally remain a promising treatment regimen for TNBC and we advocate AKT inhibitors as an alternative treatment option for TNBC.

Concurrently, AKT inhibitors typically exhibit more AEs than controls, particularly diarrhea and nausea. Hurvitz et al^[15]^ believed that active monitoring and early management of AEs in patients undergoing chemotherapy are critical. Future studies should focus on the correlation among dosage, frequency, and efficacy. To enhance the efficacy and minimize adverse reactions, more research is needed to better guide clinical practice.

Furthermore, recent studies have indicated that activation of the PI3K/AKT/mTOR pathway is linked to resistance to conventional breast cancer therapies, including endocrine therapy, HER2-targeted therapy, CDK4/6 inhibitors, PARP inhibitors, chemotherapy, and immunotherapy.^[18]^ Current preclinical and clinical evidence suggests that inhibitors of the PI3K/AKT/mTOR pathway may be used in conjunction with other anticancer therapies to circumvent drug resistance in cancer cells.^[19]^ Consequently, the most direct method to enhance the efficacy of AKT inhibitors in TNBC is to pair them with biologics that target different pathways, which help inhibit pathways that promote the survival and proliferation of cancer cells.^[20]^ Therefore, various approaches are being evaluated in clinical trials. For instance, AKT inhibitors are paired with PARP inhibitors,^[21]^ while mTORC1 inhibitors, PI3K inhibitors, dual PI3K and mTOR inhibitors, and isoform-specific PI3K inhibitors^[22]^ also show promising prospects.

However, the use of an increased number of complex compounds increases the risk of additional or cumulative toxicities. Therefore, considering the safety of AKT inhibitors, it is necessary to develop multiple combination treatment options. The outcomes of ongoing trials will determine whether AKT inhibitors will join the arsenal of treatments against TNBC. Simultaneously, concerted efforts are required to identify biomarkers of response, reduce the toxic burden of these compounds, and devise novel and effective combination strategies to successfully transition AKT inhibitors from laboratory to clinical practice.

There are also the following limitations in our study: (1) as a relatively new drug, AKT inhibitors are rarely studied and the number of relevant studies is small, which may affect the accuracy of the results; (2) the intervention drugs used in the studies were not the same, the number of included studies was small, subgroup analysis could not be performed, and there was clinical heterogeneity between studies; (3) due to the short study duration, many outcome indicators have not been reported and cannot be analyzed; therefore, its long-term efficacy needs to be further evaluated.

## 5. Conclusion

AKT inhibitors showed slightly better efficacy in the treatment of TNBC. However, further studies are needed to evaluate its long-term safety and optimal regimen, and caution should be exercised in patients with coexisting gastrointestinal disorders. The clinical characteristics of the patients and the choice of drugs should be considered on an individual basis. It is hoped that the results of this study can provide a certain reference for clinical practice and promote the improvement of the treatment effect of TNBC. Future studies should explore and identify pathways that can be targeted simultaneously with PAM pathway inhibitors to optimize TNBC outcomes.

## Author contributions

**Conceptualization:** Minghao Yang, Chunxi Wang, Guoping Chen.

**Data curation:** Minghao Yang, Chunxi Wang, Guoping Chen.

**Formal analysis:** Minghao Yang, Chunxi Wang, Guoping Chen

**Funding acquisition:** Minghao Yang.

**Investigation:** Minghao Yang, Chunxi Wang, Guoping Chen.

**Methodology:** Minghao Yang, Chunxi Wang, Guoping Chen.

**Project administration:** Minghao Yang, Chunxi Wang, Guoping Chen.

**Resources:** Minghao Yang, Chunxi Wang, Guoping Chen.

**Software:** Minghao Yang, Chunxi Wang, Guoping Chen.

**Supervision:** Minghao Yang, Chunxi Wang, Guoping Chen, Haowen Zhang, Junlong Lin.

**Validation:** Minghao Yang, Chunxi Wang, Guoping Chen.

**Visualization:** Minghao Yang, Chunxi Wang, Guoping Chen, Haowen Zhang, Junlong Lin.

**Writing – original draft:** Minghao Yang, Chunxi Wang.

**Writing – review & editing:** Minghao Yang, Chunxi Wang, Guoping Chen, Haowen Zhang, Junlong Lin.
